# Advances in research on the impact and mechanisms of pathogenic microorganism infections on pyroptosis

**DOI:** 10.3389/fmicb.2024.1503130

**Published:** 2024-12-13

**Authors:** Pan Shang, Mailin Gan, Ziang Wei, Shijie Hu, Lei Song, Jinkang Feng, Lei Chen, Lili Niu, Yan Wang, Shunhua Zhang, Linyuan Shen, Li Zhu, Ye Zhao

**Affiliations:** ^1^Farm Animal Genetic Resources Exploration and Innovation Key Laboratory of Sichuan Province, Sichuan Agricultural University, Chengdu, China; ^2^State Key Laboratory of Swine and Poultry Breeding Industry, Sichuan Agricultural University, Chengdu, China; ^3^Key Laboratory of Livestock and Poultry Multi-omics, Ministry of Agriculture and Rural Affairs, College of Animal and Technology, Sichuan Agricultural University, Chengdu, China

**Keywords:** pathogenic microorganisms, pyroptosis, inflammasome, caspase, granzyme

## Abstract

Pyroptosis, also known as inflammatory necrosis, is a form of programmed cell death characterized by the activation of gasdermin proteins, leading to the formation of pores in the cell membrane, continuous cell swelling, and eventual membrane rupture. This process results in the release of intracellular contents, including pro-inflammatory cytokines like IL-1β and IL-18, which subsequently trigger a robust inflammatory response. This process is a crucial component of the body’s innate immune response and plays a significant role in combating infections. There are four main pathways through which pathogenic microorganisms induce pyroptosis: the canonical inflammasome pathway, the non-canonical inflammasome pathway, the apoptosis-associated caspase-mediated pathway, and the granzyme-mediated pathway. This article provides a brief overview of the effects and mechanisms of pathogen infections on pyroptosis.

## 1 Introduction

**Figure 1:**
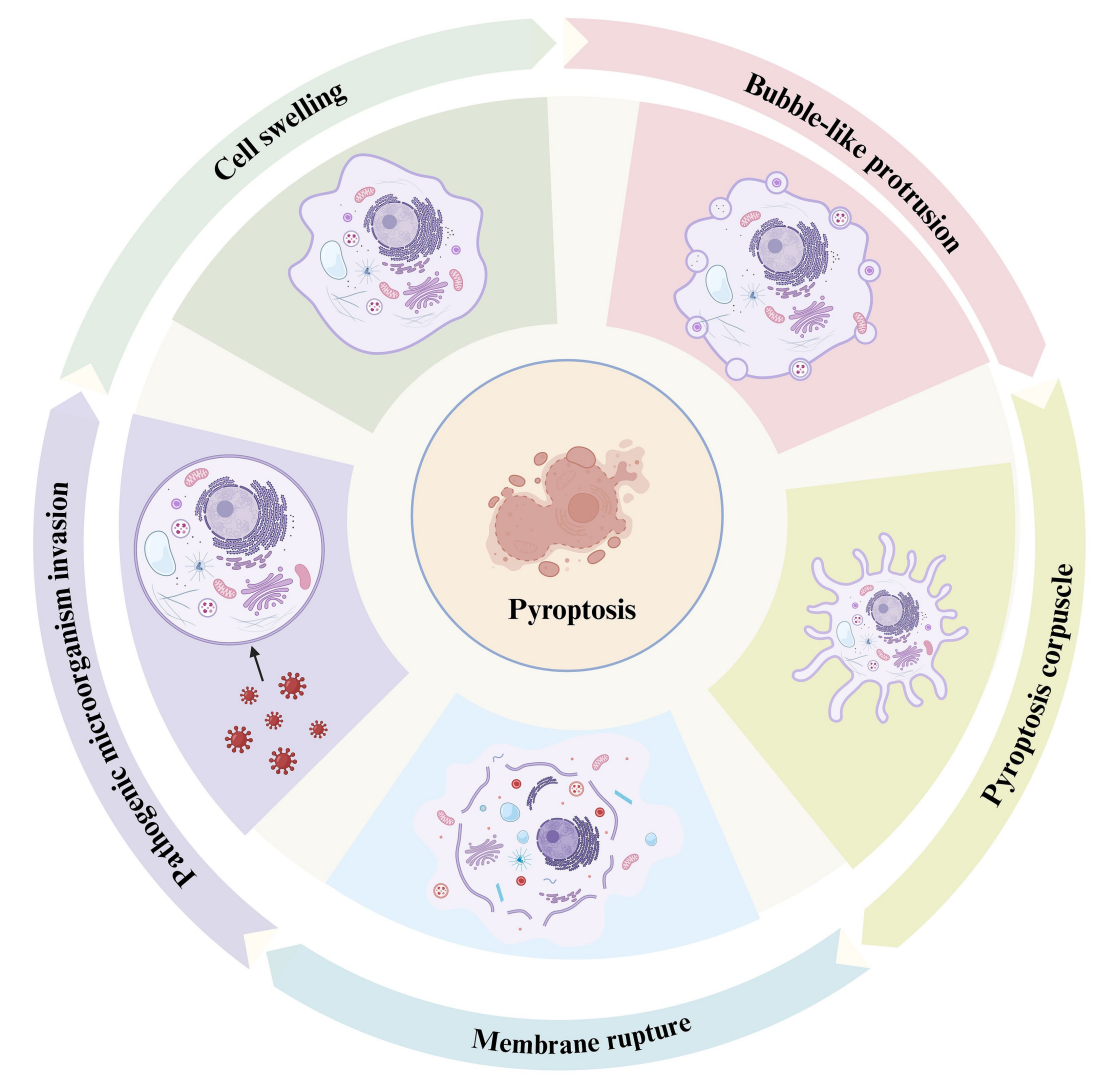
The process of pyroptosis. This diagram illustrates the sequential stages of pyroptosis, a form of programmed necrosis mediated by gasdermin. The process begins with the invasion of pathogenic microorganisms (left), during which the invading pathogens interact with the host cell, triggering an inflammatory response. Cell swelling (top left) follows as the cell undergoes structural changes in response to the infection. As the pyroptotic response intensifies, bubble-like protrusions (top right) begin to form on the cell membrane, which is a hallmark of this cell death process. Subsequently, the cell forms a pyroptosis corpuscle (bottom right), consisting of membrane-bound structures created during pyroptosis. Finally, the process concludes with membrane rupture (bottom left), where the cell membrane breaks apart, releasing pro-inflammatory cytokines such as IL-1β and IL-18, which contribute to inflammation and immune system activation.

During the growth and development of biological organisms, cell death is a ubiquitous phenomenon that plays a crucial role in the growth, development, and internal balance of multicellular organisms. Cell death can generally be categorized into two main types: programmed and non-programmed cell death. Programmed cell death includes several distinct mechanisms, such as apoptosis, necroptosis, pyroptosis, autophagy, ferroptosis, and NETosis (neutrophil extracellular trap formation). Each of these pathways plays a unique role in cellular regulation and response to physiological and pathological stimuli (Bertheloot et al., 2021; Papayannopoulos, 2018; Tang et al., 2019). Pyroptosis is a recently identified form of pro-inflammatory programmed cell death mediated by the Gasdermin protein family. Through activation by inflammatory caspases (such as caspase-1 and caspase-4/5/11), Gasdermin proteins form membrane pores that facilitate the release of numerous inflammatory mediators, triggering an amplified inflammatory cascade (Shi et al., 2017). This process is a crucial innate immune defense mechanism in the body’s response to infection (Srikanth et al., 2018). The process critically depends on the formation of plasma membrane pores by members of the gasdermin (GSDM) protein family (Kovacs and Miao, 2017). Compared to other forms of cell death, the most distinctive feature of pyroptosis is the inflammatory response. The hallmark of pyroptosis is the disruption of cell membrane integrity and the release of cellular contents into the extracellular space, which significantly distinguishes this mode of cell death from others (Chen M. et al., 2022). Initially, the most notable characteristic of pyroptotic cells during the early stages of cell death is significant cellular swelling and enlargement. Secondly, the nucleus of pyroptotic cells remains intact, characterized by chromatin condensation without DNA fragmentation (Bertheloot et al., 2021). Finally, pyroptotic cells form pores on the plasma membrane surface, exposing the inner boundary of the cell membrane to the external environment, ultimately leading to the outflow of cellular contents and the occurrence of pyroptosis (Hu et al., 2020) (Figure 1). An increasing number of studies suggest that infections by pathogenic microorganisms (including fungal toxins, bacterial toxins, and viruses) trigger a series of responses in the organism that cause damage and lead to pyroptosis (Mao et al., 2023).

## 2 The impact of pathogenic microorganisms on pyroptosis

### 2.1 The initiation mechanism of pyroptosis and the assembly of inflammasomes

Pyroptosis is a form of programmed cell death characterized by its potent pro-inflammatory effects, playing a crucial role in host defense against pathogenic infections (Elias et al., 2023). By triggering inflammatory cascades and activating the immune system, pyroptosis aids the body in rapidly countering invading pathogens (Huang et al., 2023). The initiation of pyroptosis relies on the recognition of pathogen-associated molecular patterns (PAMPs) (Kelley et al., 2019). Host pattern recognition receptors (PRRs), such as Toll-like receptors (TLRs), NOD-like receptors (NLRs), and AIM2-like receptors, detect pathogen-specific molecular patterns, subsequently triggering the assembly of inflammasomes (Fu and Wu, 2023; Olona et al., 2022; Rathinam and Fitzgerald, 2016). Upon recognizing and responding to PAMPs and endogenous damage-associated molecular patterns (DAMPs), these receptors typically engage an adaptor protein, ASC, to link upstream inflammasome sensors to downstream Caspase-1, thereby activating Caspase-1 (Dong et al., 2023; Wang et al., 2023). ASC contains both a pyrin domain (PYD) and a Caspase recruitment domain (CARD), which facilitate the oligomerization and activation of Caspase-1, driving the pyroptotic response (Diaz-Parga and de Alba, 2022). However, the assembly of certain inflammasomes, such as the NLRC4 inflammasome, can occur independently of ASC (Zhang X. et al., 2024). Instead, NLRC4 directly binds and activates Caspase-1 through its own CARD domain (Oh et al., 2023).

Outside the classic ASC-dependent pathway, Caspase-4, Caspase-5 (in humans), and Caspase-11 (in mice) play unique roles in the noncanonical pyroptosis pathway (Shi et al., 2015). These caspases can be activated by directly recognizing intracellular lipopolysaccharide (LPS) without requiring the ASC adaptor protein (Kayagaki et al., 2015). Once activated, these caspase proteins cleave Gasdermin D (GSDMD), releasing its active N-terminal fragment, which binds to membrane phospholipids, forming membrane pores (Wang et al., 2020). This pore formation facilitates the release of pro-inflammatory cytokines, such as IL-1β and IL-18, thereby triggering pyroptosis and eliciting a strong inflammatory response (Wang et al., 2021). This mechanism demonstrates that, in the noncanonical pathway, the activation of Caspase-4, -5, and -11, similar to Caspase-1 activation in the canonical pathway, initiates pyroptosis directly through GSDMD pore formation (Downs et al., 2020; Fu et al., 2024).

Moreover, recent studies have revealed that Caspase-4 and Caspase-5 in humans, as well as Caspase-11 in mice, possess functions beyond inducing pyroptosis, directly regulating specific pro-inflammatory cytokines (Exconde et al., 2023). Research indicates that Caspase-4 and Caspase-5 can cleave IL-18 directly, modulating its activity levels to fine-tune the intensity of the inflammatory response. Additionally, Caspase-4/5/11 can cleave IL-1β to produce an inactive 27-kDa fragment, inhibiting the full activation of IL-1β and, in certain contexts, preventing excessive inflammation (Shi et al., 2023). These findings expand the role of Caspase-4/5/11 within the pyroptosis pathway, indicating that they not only initiate pyroptosis through GSDMD but also regulate inflammation by directly acting on cytokines, thus forming a complex and finely tuned inflammatory regulatory network (Wang et al., 2020).

### 2.2 The pro-inflammatory and biological effects of pyroptosis and the release of cellular contents

The pro-inflammatory nature of pyroptosis largely depends on the release of various cellular contents, which include not only traditional pro-inflammatory cytokines (such as IL-1β and IL-18) but also a range of metabolites and DAMP molecules (Tsuchiya et al., 2021). These molecules play significant roles in immune response and metabolic regulation. The release of lactate dehydrogenase (LDH) indicates compromised cell membrane integrity and serves as a key marker of pyroptosis (Luo et al., 2023). Additionally, metabolites released during pyroptosis, such as ATP, uric acid, and high-mobility group box 1 (HMGB1), exert profound effects on immune regulation (Shen et al., 2021). Extracellular ATP not only attracts immune cells to the site of infection but also activates the P2X7 receptor, further promoting NLRP3 inflammasome activation and creating a positive feedback loop (Sun et al., 2023). Uric acid, as a critical DAMP, stimulates dendritic cells and macrophages to enhance antigen presentation, thereby activating the adaptive immune response (Cheng et al., 2024). Furthermore, the release of HMGB1 can bind to Toll-like receptor 4 (TLR4) and the receptor for advanced glycation end products (RAGE), promoting the production of inflammatory cytokines and exacerbating tissue damage (Chen R. et al., 2022). In addition to these molecular events, the cellular characteristics during pyroptosis, such as cell shrinkage, nuclear condensation, membrane pore formation, and vacuolization, are clearly visible under electron microscopy. These features highlight the typical morphology of nuclear shrinkage and cytoplasmic vacuolization, which further emphasize the cellular changes accompanying this process. In summary, pyroptosis plays an essential biological role in defending against pathogen invasion; however, its released contents can also lead to tissue damage and inflammation spread under pathological conditions (Mehrotra et al., 2024). These findings reveal the dual role of pyroptosis in infection and tissue damage, offering new insights for controlling infections and inflammation through regulating pyroptosis in the future.

### 2.3 The Gasdermin family proteins and their role in pyroptosis

The Gasdermin family proteins (GSDMs) are key executioners in cellular pyroptosis, primarily including GSDMA, GSDMB, GSDMC, GSDMD, GSDME, and PJVK (also known as DFNB59) (Ding et al., 2016). Except for PJVK (DFNB59), structurally, these proteins contain both an N-terminal domain (responsible for pore formation) and a C-terminal domain (inhibiting N-terminal activity), a configuration that plays a critical role in their functional activation and regulation (Bordon, 2016). GSDMD is the central executor in pyroptosis (Shi et al., 2017). Upon pathogen invasion, it is cleaved by Caspase-1 and the noncanonical Caspases-4, -5, or -11, releasing the N-terminal fragment, which embeds in the cell membrane to form pores and release pro-inflammatory cytokines IL-1β and IL-18, thereby initiating an immune response (Zhu et al., 2024).

In mammals, other Gasdermin proteins also exhibit diverse immune functions (LaRock et al., 2022). For example, GSDMA is primarily expressed in epithelial tissues and may play a role in inflammation regulation and cancer suppression (Billman et al., 2024; Deng et al., 2022). GSDMB, which shows increased expression in inflammatory diseases and certain cancers, can be activated by granzyme A secreted by immune cells (Rana et al., 2022; Zhou et al., 2020). This activation directly mediates pyroptosis in tumor cells, contributing to the anti-tumor immune response (Kong et al., 2023). GSDMC is activated in acidic or tumor microenvironments, triggering pyroptosis through pore formation to help limit tumor spread (Hou et al., 2020; Zhang et al., 2021). GSDME, mainly activated by Caspase-3, increases cancer cell sensitivity to treatment by promoting pyroptosis during chemotherapy-induced cell death, thus enhancing cell lysis and exhibiting anti-tumor effects (Jiang M. et al., 2020; Wei Y. et al., 2023). Although the specific role of PJVK (DFNB59) in pyroptosis remains unclear, its involvement in auditory protection and other cellular stress responses provides clues to a potential role in the regulation of cell death (Collin et al., 2007; Delmaghani et al., 2006).

In contrast, Gasdermin proteins in lower eukaryotes generally lack this level of specialized functional differentiation. Although GSDMs in these organisms also possess N-terminal and C-terminal domains, their activation relies more on direct regulation by intracellular signaling molecules rather than Caspase cleavage (Li et al., 2024). As a result, these GSDMs primarily function in basic immune responses and do not exhibit the specialized roles seen in mammals, such as GSDMA’s involvement in cancer suppression and GSDMB’s regulation of inflammatory processes. Recent studies have further revealed that, beyond the traditional Caspase-dependent activation pathway, certain lower eukaryotes and even some mammals possess Caspase-independent mechanisms for activating GSDMs (Angosto-Bazarra et al., 2022; Jiang S. et al., 2020; Qin et al., 2023). In these cases, intracellular signaling molecules induce conformational changes in GSDMs, activating the pore-forming capability of their N-terminal domains and triggering membrane pore formation and pyroptosis. This alternative mechanism offers new insights into the diversity of GSDM regulation across species and may represent a unique adaptive advantage in pathogen defense, especially in lower organisms.

### 2.4 Pyroptotic pathways induced by pathogenic microorganisms

Pathogenic microorganisms activate Gasdermin family proteins through various mechanisms, triggering pyroptotic immune responses in the host and leading to intense inflammation (Rao et al., 2022). Fungal toxins, bacterial toxins, and viral proteins each activate Caspase proteins and cleave Gasdermin proteins via specific pathways and mechanisms, forming pores in the cell membrane, releasing pro-inflammatory factors, and ultimately inducing pyroptosis. For example, fungal toxins such as aflatoxin A and fumonisin B1 activate Caspases through the NLRP3 or AIM2 inflammasomes, cleaving GSDMD to form membrane pores and provoke a strong inflammatory response (Table 1) (Mao et al., 2023; Zhang et al., 2019). Bacterial toxins, like lipopolysaccharide (LPS), Mycobacterium protein Ms-PE-PGRS19, and Legionella thyA, activate the noncanonical inflammasome pathway by directly binding to Caspase-4, Caspase-5, or Caspase-11, leading to GSDMD cleavage, formation of membrane pores, and the release of IL-1β and IL-18, which further strengthen host defenses (Table 2) (Case et al., 2013; Ma et al., 2021; Qian et al., 2022; Zhong et al., 2020).

**Table 1:** Effects of mycotoxins on cell pyroptosis.

Mycotoxins	*In vivo*	Experimental dose	*In vivo*	Experimental dose	Caspase	GSDM	Pyroptosis	References
AFB1	Mice	1 mg/kg	HepaRG	0.05, 0.25 μM	Caspase-1	GSDMD	Plasma Membrane Rupture	Zhang et al. (2019)
FB1	Mice	0.1, 1 mg/kg	IPEC-J2	2, 4, 8 μM	Caspase-1	GSDMD	Plasma Membrane Rupture	Mao et al. (2022b)
OTA	Mice	1, 2 mg/kg	MDCK	0.5, 1, 2 μg/mL	Caspase-1	GSDMD	Plasma Membrane Rupture	Li et al. (2021)
PAT	Mice	0.5, 1, 2 mg/kg	HepG2	2, 4, 8 μM	Caspase-1	GSDMD	Plasma Membrane Rupture	Chu et al. (2021)
DON+FB1	Mice	25 μg/kg DON+100 μg/kg FB1	IPEC-J2	1 μM DON+4 μM FB1	Caspase-1	GSDMD	Plasma Membrane Rupture	Mao et al. (2023)
ZEA	–	–	INS-1	50, 100, 200 μM	Caspase-1	GSDMD	Plasma Membrane Rupture	Wang et al. (2019)
DON	Mice	4, 5, 6 mg/kg	HepaRG	32, 64 μM	Caspase-3	GSDME	Plasma Membrane Rupture	Mao et al. (2022a)

–, Number of genes unknown.

**Table 2:** Effects of bacterial toxins on pyroptosis in cells.

Bacterial toxins	*In vivo*	Experimental dose	*In vivo*	Experimental dose	Caspase	GSDM	Pyroptosis	References
LPS	–	–	BEECs	3, 10, 30 μg/mL	Caspase-1, 4	GSDMD	Plasma Membrane Rupture	Ma et al. (2021)
Ms-PE-PGRS19	–	–	J774A.1	1 MOI	Caspase-1, 11	GSDMD	Plasma Membrane Rupture	Qian et al. (2022)
thyA	–	–	MΦ	10 MOI	Caspase-1, 4	GSDMD	Plasma Membrane Rupture	Case et al. (2013)
EPEC	–	–	IEC	1 MOI	Caspase-1, 4	GSDMD	Plasma Membrane Rupture	Zhong et al. (2020)

–, Number of genes unknown.

Studies have shown that guanylate-binding proteins (GBPs) play a crucial role in LPS-induced noncanonical inflammasome activation (Fisch et al., 2019; Meunier et al., 2015). As a family of interferon-inducible GTPases, GBPs enhance the host’s antibacterial capacity by promoting intracellular LPS delivery and Caspase activation during bacterial infections. Viruses, on the other hand, induce pyroptosis through multiple mechanisms (Dickinson et al., 2023; Enosi et al., 2023; Wandel et al., 2020). For instance, the spike protein of SARS-CoV-2 promotes ROS production and calcium influx to activate the NLRP3 inflammasome, subsequently activating Caspase-1 and cleaving GSDMD to trigger pyroptosis (Soares et al., 2023). Additionally, infection by some viruses, such as single-stranded RNA viruses and double-stranded DNA viruses, can prompt immune cells like T cells and NK cells to secrete granzyme A, directly activating GSDMB and enhancing antiviral immune responses (Table 3) (Kong et al., 2023; Zhong et al., 2023; Zhou et al., 2020).

**Table 3:** Effects of the virus on cell pyroptosis.

Virus	Type	*In vivo*	Experimental dose	*In vivo*	Experimental dose	Caspase	GSDM	Pyroptosis	References
JEV	Single-Stranded RNA Virus	–	–	PK15	1 MOI	Caspase-1	GSDMD	Plasma Membrane Rupture	Xu et al. (2023)
ECHO-11	Single-Stranded RNA Virus	–	–	THP-1	0.1, 0.5, 1 MOI	Caspase-1	GSDMD	Plasma Membrane Rupture	Wang et al. (2022)
TGEV	Single-Stranded RNA Virus	–	–	ST	1 MOI	Caspase-1	GSDMD	Plasma Membrane Rupture	Zhao L. et al. (2022)
SFTSV	Single-Stranded Negative-Sense RNA Virus	Mice	1 × 10^5^ PFU	PBMC	0.1, 1, 2 MOI	Caspase-1	GSDMD	Plasma Membrane Rupture	Li et al. (2022)
DBV	Double-Stranded RNA Virus	–	–	THP-1	0.1, 1 MOI	Caspase-1	GSDMD	Plasma Membrane Rupture	Yu et al. (2023)
HuNOV	Double-Stranded RNA Virus	–	–	Caco2	2 μg	Caspase-1	GSDMD	Plasma Membrane Rupture	Chen et al. (2023)
PRRSV	Single-Stranded Positive-Sense RNA Virus	Piglet	4.4 × 10^5^TCID _50_/mL	PAM	0.1, 1, 10 MOI	Caspase-1	GSDMD	Plasma Membrane Rupture	He et al. (2022)
CVB3, EV71	Single-Stranded Positive-Sense RNA Virus	Mice	1 × 106TCID _50_/μL	HeLa	1 MOI	Caspase-1	GSDMD	Plasma Membrane Rupture	Wang et al. (2018)
CSFV	Single-Stranded Positive-Sense RNA Virus	–	–	PBMC	0.1, 1 MOI	Caspase-1	GSDMD	Plasma Membrane Rupture	Fan et al. (2018)
CSFV	Single-Stranded Positive-Sense RNA Virus	Piglet	10^5^TCID_50_	–	–	Caspase-1	GSDMD	Plasma Membrane Rupture	Yuan et al. (2018)
EV-A71	Single-Stranded Positive-Sense RNA Virus	–	–	SH-SY5Y	1 MOI	Caspase-1	GSDMD	Plasma Membrane Rupture	Zhu et al. (2018)
SARS-COV-2	Single-Stranded Positive-Sense RNA Virus	–	–	Calu-3	0.1 MOI	Caspase-1	GSDMD	Plasma Membrane Rupture	Soares et al. (2023)
DHAV-1	Single-Stranded Positive-Sense RNA Virus	–	–	DEFs	0.1, 1, 10 MOI	Caspase-3	GSDME	Plasma Membrane Rupture	Wang et al. (2024)
ZIKV	Single-Stranded Positive-Sense RNA Virus	–	–	JEG-3	0.1 MOI	Caspase-3	GSDME	Plasma Membrane Rupture	Zhao Z. et al. (2022)
SVV	Single-stranded Negative-sense RNA Virus	–	–	SK6	1 MOI	Caspase-1	GSDMD	Plasma Membrane Rupture	Wen et al. (2021)
RABV	Single-stranded Negative-sense RNA Virus	Mice	0.1 mL, 0.5 mL	–	–	Caspase-1	GSDMD	Plasma Membrane Rupture	Yu D. et al. (2024)
RSV	Single-stranded Negative-sense RNA Virus	–	–	THP-1	1 MOI	Caspase-1	GSDMD	Plasma Membrane Rupture	Bedient et al. (2020)
VSV	Single-stranded Negative-sense RNA Virus	–	–	A549, EMT-6, B16	1 MOI	Caspase-3	GSDME	Plasma Membrane Rupture	Lin et al. (2022)
VSV	Single-stranded Negative-sense RNA Virus	–	–	NHEK	10 MOI	Caspase-3	GSDME	Plasma Membrane Rupture	Orzalli et al. (2021)
H1N1	Single-stranded Negative-sense RNA Virus	Mice		MDCK, BEAS-2B, A549	2, 4 LD50	Caspase-3	GSDME	Plasma Membrane Rupture	Wei Z. et al. (2023)
H7N9	Single-stranded Negative-sense RNA Virus	Mice		HPAE, HPAE II, A549, Mle12	105.5 EID_50_	Caspase-3	GSDME	Plasma Membrane Rupture	Wan et al. (2022)
IAV	Single-stranded Negative-sense RNA Virus	–	–	BMDM	2 MOI	Caspase-8	GSDMD	Plasma Membrane Rupture	Lei et al. (2023)
HBV	Double-stranded DNA Virus	–	–	HK-2	–	Caspase-1	GSDMD	Plasma Membrane Rupture	Feng et al. (2023)
HSV-2	Double-stranded DNA Virus	–	–	SH-SY5Y	1, 3 MOI	Caspase-3	GSDME	Plasma Membrane Rupture	Ren et al. (2023)
CAV	Double-stranded DNA Virus	–	–	HCT116	100 MOI	Caspase-3	GSDME	Plasma Membrane Rupture	Liu et al. (2022)
ORFV	Double-stranded DNA Virus	Mice	1 × 10^4^–1 × 10^5^TCID _50_	NCI-H226, ACHN, EMT6, CT26	1 MOI	Caspase-3	GSDME	Plasma Membrane Rupture	Lin et al. (2023)
HAdV	Double-stranded DNA Virus	–	–	THP-1	100 MOI	Caspase-4, 5	GSDMD	Plasma Membrane Rupture	Li et al. (2023)
PRV	Double-stranded DNA Virus	–	–	PAM	1, 3, 5 MOI	Caspase-1	GSDMD	Plasma Membrane Rupture	Zhang et al. (2023)

–, Number of genes unknown.

These findings demonstrate that diverse pathogens effectively activate pyroptosis through multiple pathways, significantly amplifying the host inflammatory response and providing a powerful immune mechanism for defending against infection.

### 2.5 Pathogen strategies for manipulating pyroptosis to evade host immunity

Pyroptosis is a critical immune defense mechanism employed by the host to counter pathogen invasion. However, certain pathogens have evolved complex strategies to disrupt this process, thereby evading immune surveillance and prolonging their survival within the host (Van Hauwermeiren and Lamkanfi, 2022). For instance, Salmonella secretes the effector protein SopB, which inhibits inflammasome assembly, preventing Caspase-1 activation and ultimately blocking GSDMD cleavage and pore formation, thereby suppressing the host’s pyroptotic response (Meng et al., 2024). Enteropathogenic *Escherichia coli* (EPEC), on the other hand, employs its 3C protease to directly cleave host GSDMD, halting the initiation of pyroptosis and downstream inflammatory responses (Zhong et al., 2020). These strategies enable bacteria to maintain prolonged survival within the host environment.

Viruses have also developed diverse mechanisms to manipulate the host’s pyroptotic pathways. Epstein–Barr virus (EBV), for example, uses its BHRF1 protein to bind GSDME, directly inhibiting pore formation on the host cell membrane and thereby reducing cell lysis and the inflammatory response, which supports the virus’s long-term persistence (Yiu et al., 2023). In contrast, influenza virus adopts an opposite approach, actively inducing pyroptosis in host cells to accelerate its own spread. SARS-CoV-2 has been found to interfere with host inflammasome activation through its ORF3a and Nsp1 proteins, blocking pyroptosis and enhancing viral replication (Soares et al., 2023). These diverse manipulation strategies highlight how various pathogens utilize specific molecular mechanisms to regulate host pyroptosis, thereby gaining significant survival advantages during infection.

In addition to pathogen-mediated regulation of pyroptosis, host proteins also play a role in fine-tuning pyroptosis to balance the intensity of the inflammatory response. Recent studies have identified that NINJ1 (Nerve Injury-Induced Protein 1) is crucial in the downstream process of GSDMD-mediated pore formation (David et al., 2024). NINJ1 stabilizes these membrane pores, promoting the release of cellular contents and amplifying the inflammatory cascade triggered by pyroptosis (Degen et al., 2023; Kayagaki et al., 2021). This process not only helps amplify the host immune response at the infection site but may also provide sustained pro-inflammatory signals that guide nearby immune cells to the infection source, further strengthening the host’s defense strategy.

In addition, the host regulates pyroptosis by modifying Gasdermin family proteins (Sun and Hornung, 2024). Recent research indicates that palmitoylation of GSDMD directly influences its membrane-binding ability (Balasubramanian et al., 2024). This modification enhances the targeting and pore-forming efficiency of GSDMD on the membrane, thereby ensuring the rapid release of pro-inflammatory cytokines such as IL-1β and IL-18 (Du et al., 2024; Yu T. et al., 2024; Zhang N. et al., 2024). This regulatory mechanism of palmitoylation demonstrates that the host can finely control pyroptosis during pathogen defense by both stabilizing pores via NINJ1 and optimizing GSDMD membrane binding through palmitoylation (Lin et al., 2024). This dual approach provides effective immune defense during the early stages of infection while minimizing excessive tissue damage. Such regulatory insight offers a new perspective on the role of pyroptosis in infectious diseases, paving the way for a deeper understanding in future research.

Overall, as a form of programmed cell death with potent pro-inflammatory effects, pyroptosis plays a crucial immune defense role in the host’s response to pathogen invasion (Newton et al., 2024). Various pathogens induce pyroptosis in host cells through multiple pathways, activating Gasdermin family proteins, assembling inflammasomes, and releasing a range of pro-inflammatory molecules. To evade host immunity, certain pathogens have evolved strategies to inhibit or manipulate pyroptosis activation, enabling immune evasion and more efficient dissemination (Hu et al., 2024). Beyond its role in infection defense, the biological functions of pyroptosis include potential tissue damage and pathological responses due to the release of inflammatory contents, highlighting its dual role in infection, immune response, and tissue injury (Vasudevan et al., 2023). Further research into the molecular mechanisms of pyroptosis and the ways pathogens regulate it could provide promising new approaches for the prevention and treatment of infectious diseases.

## 3 Mechanisms of pyroptosis induced by pathogenic microorganism infections

### 3.1 The classical pyroptosis pathway and its upstream regulatory mechanisms

**Figure 2:**
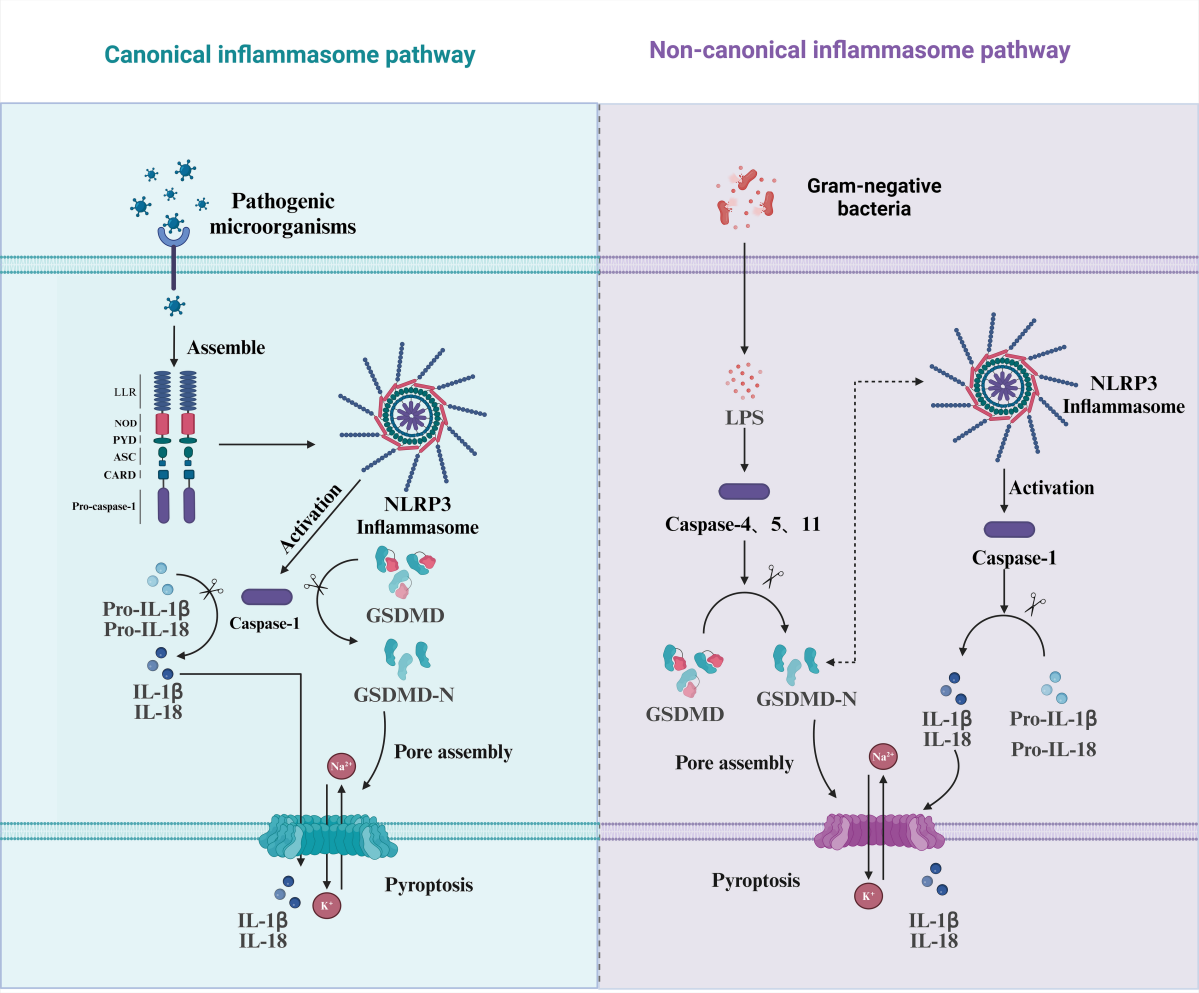
Pyroptosis mediated by the canonical and non-canonical inflammasome pathways. This diagram illustrates the two major pathways involved in the induction of pyroptosis: the canonical inflammasome pathway and the non-canonical inflammasome pathway. In the canonical pathway (left), pathogenic microorganisms trigger the assembly of the NLRP3 inflammasome by activating pattern recognition receptors such as NOD, PYD, ASC, and CARD. This leads to the activation of Caspase-1, which cleaves pro-inflammatory cytokines (Pro-IL-1β and Pro-IL-18) into their active forms (IL-1β and IL-18) and also activates GSDMD, releasing its N-terminal domain (GSDMD-N), which then assembles pores in the membrane, culminating in pyroptosis. In the non-canonical pathway (right), gram-negative bacteria release LPS, which is recognized by Caspase-4/5/11. This activation also leads to the cleavage of GSDMD, resulting in the release of its active N-terminal domain (GSDMD-N) and pore formation. In this pathway, Caspase-1 is also activated but is involved in cleaving Pro-IL-1β and Pro-IL-18, promoting further inflammation and the onset of pyroptosis. Both pathways culminate in membrane rupture and the release of inflammatory cytokines, playing a crucial role in the host’s immune defense response.

The canonical inflammasome pathway was the first to be identified in the process of pyroptosis induced by pathogenic microorganisms. Inflammasomes are multiprotein complexes assembled in response to pathogen-associated molecular patterns (PAMPs) or damage-associated molecular patterns (DAMPs). Typically, inflammasomes consist of cytoplasmic pattern recognition receptors (PRRs), apoptosis-associated speck-like proteins containing a caspase recruitment domain (ASC), and inflammatory caspases (Zheng et al., 2020). The most common PRRs include NOD-like receptors (NLRs), such as NLRP1, NLRP3, and NLRC4, as well as AIM2 and Pyrin (Burdette et al., 2021). The NLRP3 is composed of PYD, NOD, and LRR domains (Hu and Chai, 2023). The PYD is responsible for binding with ASC, the NOD is involved in ATP-dependent signaling activation, the LRR is tasked with ligand recognition and self-inhibition, and the CARD is involved in recruiting the precursor of Caspase-1 (Pro-Caspase-1) (Vande and Lamkanfi, 2024). Under the stimulation of PRRs, Pro-Caspase-1 is recruited directly by PRRs with a CARD or indirectly through ASC, assembling a Caspase-1-dependent inflammasome. Subsequently, Caspase-1 is activated through self-cleavage (Fu and Wu, 2023). Once activated, Caspase-1 not only cleaves the inactive precursors of IL-1β and IL-18 but also cleaves GSDMD, releasing the N-terminal fragment of GSDMD. This N-terminal fragment binds to phospholipids in the cell membrane to form membrane pores, thereby facilitating the release of the inflammatory cytokines IL-1β and IL-18 (Yuan and Ofengeim, 2024) (Figure 2).

#### 3.1.1 Activation of pyroptosis through the NLRP3 inflammasome pathway

Pathogens such as viruses and fungi activate the NLRP3 inflammasome through various mechanisms, thereby inducing pyroptosis. As a central regulator of inflammatory responses, the NLRP3 inflammasome can be activated through various mechanisms by different pathogens, ultimately leading to the onset of pyroptosis. AFB1, for instance, promotes the assembly and activation of the NLRP3 inflammasome by dephosphorylating cyclooxygenase-2 (COX-2), altering its activity, and indirectly facilitating pyroptosis (Zhang et al., 2019). Autophagy helps remove intracellular damaged structures that can lead to the overactivation of the NLRP3 inflammasome. During FB1 intoxication, when mTOR (mammalian target of rapamycin) activity is excessively high, autophagy is inhibited, resulting in the accumulation of reactive oxygen species (ROS). This excessive ROS triggers the overactivation of the NLRP3 inflammasome, ultimately leading to pyroptosis (Mao et al., 2022b). NF-κB is a key transcription factor that regulates the expression of various pro-inflammatory genes. During ZEA intoxication, activation of the NF-κB signaling pathway promotes NLRP3 inflammasome-dependent pyroptosis (Wang et al., 2019). During ECHO-11 (Echovirus 11) infection, the viral 2B protein serves as a critical mediator of pyroptosis by directly interacting with the host NLRP3 protein, facilitating the assembly and activation of the NLRP3 inflammasome, ultimately triggering pyroptosis (Wang et al., 2022). In the case of HBV (Hepatitis B virus) infection, the viral HBx protein promotes pyroptosis through upregulation of PLA2R (M-type phospholipase A2 receptor), which subsequently activates the NLRP3 inflammasome, leading to pyroptosis (Feng et al., 2023). For HuNoV (Human Norovirus), the viral non-structural protein P22 plays a central role in pyroptosis induction by activating the NLRP3 inflammasome, which then initiates the pyroptotic pathway (Chen et al., 2023). Overall, various pathogens activate the NLRP3 inflammasome through distinct mechanisms, ultimately triggering pyroptosis. This highlights the NLRP3 inflammasome as a critical regulator of pathogen-induced inflammatory and pyroptotic responses.

#### 3.1.2 Pyroptosis is induced or inhibited through interactions between apoptosis-related proteins or specific viral proteins and host proteins

Some pathogens regulate host apoptosis-related proteins or utilize their own viral-encoded proteins to interact with host proteins, either activating or inhibiting pyroptosis. These mechanisms can promote pathogen replication and help evade the host’s immune defense. BAK (BCL-2 antagonist/killer) is a pro-apoptotic protein from the BCL-2 family that plays a crucial role in both apoptosis and pyroptosis. During JEV (Japanese Encephalitis Virus) infection, the transcription of BAK is upregulated, enhancing its function on the mitochondrial outer membrane, increasing membrane permeability, and triggering downstream pyroptosis signaling pathways (Xu et al., 2023). Upon EBV (Epstein–Barr virus) infection, the viral BILF1 protein binds to the host’s mitochondrial antiviral signaling protein (MAVS), triggering its UFMylation. This modification suppresses NLRP3 inflammasome activation, enabling EBV to evade the pyroptosis-based immune response. By inhibiting the inflammatory process and blocking the release of pro-inflammatory cytokines, this mechanism aids viral persistence and replication within the host (Yiu et al., 2023). In the case of SFTS (Severe Fever with Thrombocytopenia Syndrome), the non-structural protein NSs associates with and co-localizes alongside NLRP3, facilitating its assembly and activation. Additionally, the N-terminal region of NSs (amino acids 1–66) enhances the expression of pyroptosis-related genes, which activates subsequent signaling cascades and triggers pyroptosis (Gao et al., 2022). Ultimately, these pathogens regulate pyroptosis through intricate interactions with host proteins, allowing them to enhance their survival and replication or evade the host’s immune surveillance.

#### 3.1.3 Regulation of intracellular structures or metabolic and stress-related pathways induces pyroptosis

The Trans-Golgi Network (TGN) is a critical part of the Golgi apparatus, responsible for sorting and transporting proteins and lipids, ensuring the proper function and metabolic balance of the intracellular membrane system. Following PRRSV (Porcine Reproductive and Respiratory Syndrome Virus) infection, the structure of the TGN is disrupted. The dispersed TGN, acting through phosphatidylinositol-4-phosphate (PI4P) as a scaffold, activates the NLRP3 inflammasome, which subsequently upregulates pyroptosis-related factors, activates downstream signaling pathways, and ultimately induces pyroptosis (He et al., 2022). The RIPK3-MLKL pathway, which includes receptor-interacting serine/threonine-protein kinase 3 (RIPK3) and mixed lineage kinase domain-like pseudokinase (MLKL), is essential for regulating necroptosis, especially in response to viral infections or cellular stress. By promoting pore formation in the cell membrane through MLKL, this pathway leads to cell death and inflammation. Upon infection with RSV (Respiratory Syncytial Virus), the activation of RIPK3-MLKL upregulates pyroptosis-related factors, thereby inducing pyroptosis (Bedient et al., 2020). SREBP1 (Sterol Regulatory Element-Binding Protein 1) and SREBP2 (Sterol Regulatory Element-Binding Protein 2) are key transcription factors that regulate cellular lipid metabolism and belong to the SREBP family. SARS-CoV-2 (Severe Acute Respiratory Syndrome Coronavirus 2) activates the SREBP1 and SREBP2 pathways, leading to lipid metabolism imbalance and mitochondrial stress in host cells. This activation subsequently triggers the NLRP3 inflammasome, initiating downstream signaling pathways and inducing pyroptosis (Soares et al., 2023). In essence, these pathogens activate the NLRP3 inflammasome through a variety of distinct mechanisms, leading to pyroptosis and amplifying the inflammatory response. This underscores the diversity of their pathogenic pathways.

To summarize, various pathogens activate the NLRP3 inflammasome and induce pyroptosis through distinct molecular mechanisms and signaling pathways. These include direct interactions with inflammasome proteins, regulation of apoptosis-related proteins, disruption of cellular structures, and the induction of metabolic or stress imbalances. While the pathways differ, they all converge on pyroptosis-related inflammatory responses, significantly influencing the host’s immune defense. Gaining a deeper understanding of these mechanisms not only sheds light on the intricate regulation of pathogen infections but also offers a theoretical foundation for developing targeted therapies to inhibit inflammasome activation.

### 3.2 Non-canonical pyroptosis pathway and its upstream regulatory mechanisms

The non-canonical pyroptosis pathway is directly recognized and activated by Caspase-4, Caspase-5, and Caspase-11, which bind to LPS in the cell walls of Gram-negative bacteria and activate their own protease activity (Shi et al., 2014). Activated Caspase-4, Caspase-5, and Caspase-11 can also cleave GSDMD in a manner similar to Caspase-1, resulting in the perforation of the cell membrane (Kayagaki et al., 2015; Shi et al., 2014). Once activated, Caspase-4, Caspase-5, and Caspase-11 can directly cleave GSDMD protein, with the GSDMD-N terminus oligomerizing to form pores in the cell membrane (Ruhl and Broz, 2015). Unlike the canonical pyroptosis pathway, where Caspase-1 activates and cleaves the pro-inflammatory factors pro-IL-18 and pro-IL-1β, the non-canonical caspases, Caspase-4, Caspase-5, and Caspase-11, exhibit distinct roles. These caspases directly cleave IL-18, and while they also cleave pro-IL-1β, they generate a non-functional 27-kDa fragment of IL-1β, preventing its full activation and reducing the intensity of the inflammatory response (Exconde et al., 2023). However, the formation of pores in the cell membrane allows the flow of potassium and sodium ions, which can activate the formation of the NLRP3 inflammasome. The activated NLRP3 inflammasome then activates Caspase-1, which cleaves pro-IL-18 and pro-IL-1β, releasing them into the extracellular space to recruit more inflammatory mediators and amplify the inflammatory response (Beckwith et al., 2020; Matikainen et al., 2020) (Figure 2).

#### 3.2.1 Direct activation of the non-canonical inflammasome pathway through Caspase-11/Caspase-4/Caspase-5

This class of pathogens induces pyroptosis by directly activating Caspase-11 (in mice) or Caspase-4 and Caspase-5 (in humans), initiating the non-canonical inflammasome pathway. These pathogens utilize specific molecular mechanisms to regulate the host’s recognition of pathogen-associated molecular patterns, such as LPS or other bacterial components, and activate the inflammasome, triggering the release of pro-inflammatory cytokines and pyroptotic responses. The PE/PPE protein family, unique to *Mycobacterium tuberculosis*, typically resides in the bacterial cell wall or is secreted into host cells, playing a crucial role in pathogen-host interactions and contributing to the virulence of *M. tuberculosis*. Following *M. tuberculosis* infection, the PE/PPE protein family modulates the host’s inflammatory response by promoting the expression of pro-inflammatory cytokines. This leads to the activation of inflammatory molecules such as Caspase-11 via the non-canonical pathway, ultimately inducing pyroptosis (Qian et al., 2022). During EPEC (enteropathogenic *E. coli*) infection, the aggregation of the bacterial protein Tir (Translocated Intimin Receptor) triggers a rapid influx of calcium ions, leading to LPS internalization and Caspase-4 activation. This activates downstream signaling pathways, ultimately resulting in pyroptosis. Knockdown of Caspase-4 or GSDMD, inhibition of the translocated effector protein NleF, or chelation of extracellular calcium ions can all suppress EPEC-induced cell death (Zhong et al., 2020). NF-κB is a key transcription factor that regulates the expression of various pro-inflammatory genes. Following HAdV (Human Adenovirus) infection, activation of the NF-κB signaling pathway induces the activation of non-canonical inflammasomes and triggers pyroptosis. Inhibition of the NF-κB pathway significantly reduces HAdV-induced non-canonical inflammasome activation and pyroptosis. Additionally, knocking down Caspase-4 and Caspase-5 expression greatly diminishes HAdV-induced pyroptosis (Li et al., 2023). Fundamentally, these pathogens initiate the non-canonical inflammasome pathway by directly activating Caspase-11 or Caspase-4/5, resulting in pyroptosis and pro-inflammatory responses. This emphasizes their pivotal role in pathogen-host interactions and sheds light on their crucial pathogenic mechanisms.

#### 3.2.2 Indirect activation of the inflammasome through cellular stress, ion flux, or protein interactions

This group of pathogens indirectly activates the inflammasome through diverse upstream signaling pathways, such as cellular stress responses, ion flux regulation, or direct interactions with inflammasome-associated proteins, ultimately leading to pyroptosis. Following infection by *Legionella pneumophila* (thyA), its Dot/Icm secretion system can activate Caspase-1 through an NAIP5/NLRC4-independent pathway, triggering downstream signaling cascades and leading to pyroptosis (Case et al., 2013). During IAV (Influenza A virus) infection, the cell death regulator MLKL facilitates potassium ion efflux, partially activating the NLRP3 inflammasome. This activation triggers downstream signaling pathways, ultimately leading to pyroptosis (Lei et al., 2023). At a fundamental level, these pathogens activate the inflammasome and induce pyroptosis through indirect mechanisms, such as cellular stress, ion flux, or protein interactions. This showcases the diverse strategies pathogens use to evade immune responses or facilitate infection.

Overall, different pathogens activate the inflammasome and induce pyroptosis through various mechanisms. Some directly trigger Caspase-11, Caspase-4, or Caspase-5 to initiate the non-canonical inflammasome pathway, leading to a strong pro-inflammatory response. Others indirectly activate the inflammasome via cellular stress, ion flux, or specific protein interactions. These diverse mechanisms demonstrate how pathogens precisely regulate host cell death pathways during infection, allowing them to evade immune defenses or exacerbate inflammation. This understanding provides critical insights for exploring pathogenic mechanisms and developing targeted therapies.

### 3.3 Apoptotic caspase-mediated pyroptosis pathway and its upstream regulatory mechanisms

**Figure 3:**
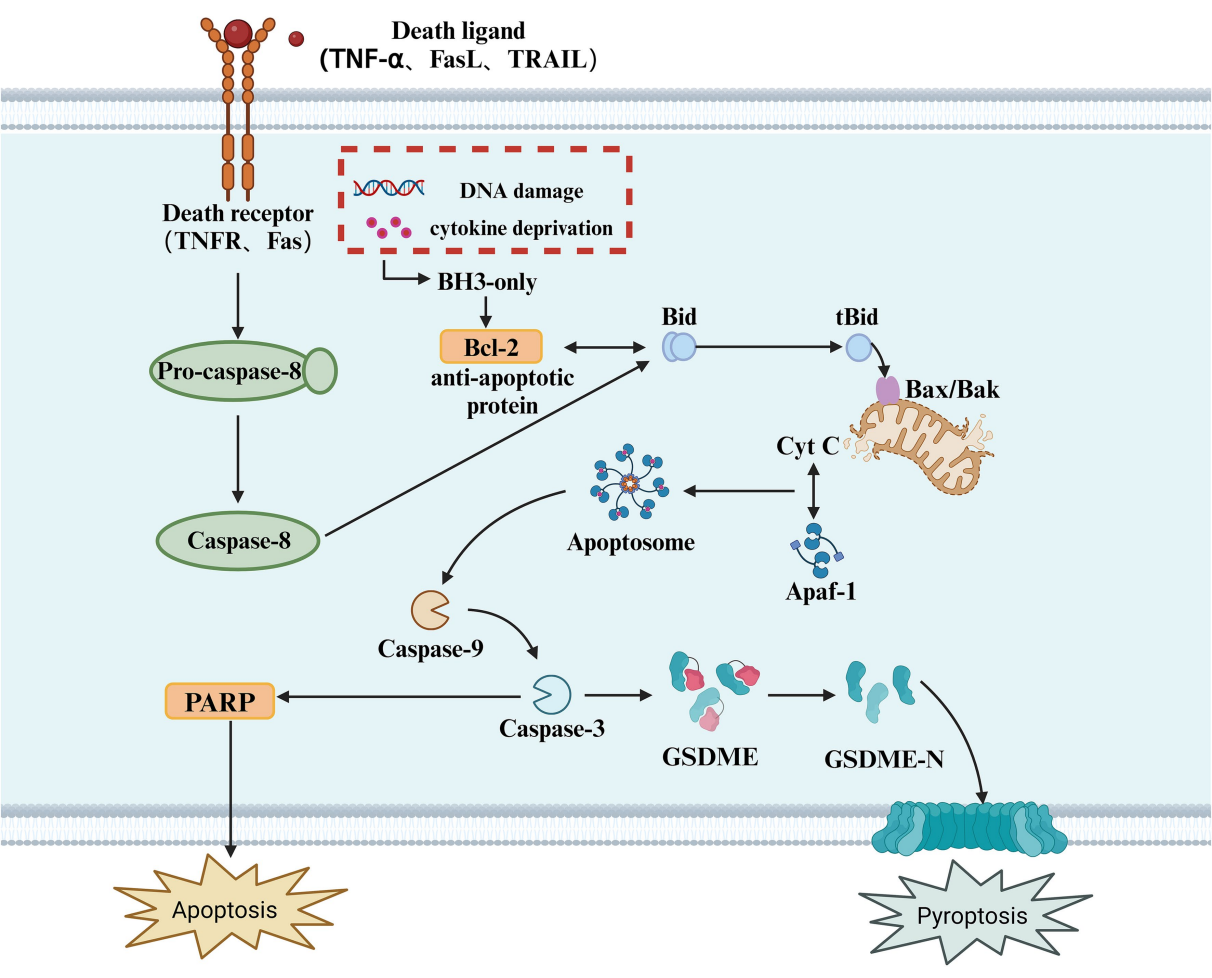
Apoptotic caspase-mediated pyroptosis. This diagram illustrates the apoptotic signaling pathway, which can also lead to pyroptosis. The process is initiated by the binding of death ligands (such as TNF-α, FasL, or TRAIL) to their respective receptors (TNFR, Fas), triggering the activation of Caspase-8. In response to cellular stress, such as DNA damage or cytokine deprivation, BH3-only proteins are activated, which in turn modulate anti-apoptotic proteins like Bcl-2. The activation of Caspase-8 leads to the cleavage of Bid, generating tBid, which translocates to the mitochondria and interacts with Bax/Bak, facilitating the release of Cyt C from the mitochondria. This activation leads to the formation of the Apoptosome, which activates Caspase-9, and subsequently Caspase-3. Caspase-3 cleaves GSDME, releasing its active N-terminal fragment (GSDME-N), which forms pores in the cell membrane, ultimately resulting in pyroptosis. Additionally, in this pathway, PARP cleavage is also a key event in apoptosis, with its activation promoting cellular disassembly. Pyroptosis, as an inflammatory form of cell death, results from caspase activation, which causes the release of pro-inflammatory cytokines and the rupture of the cell membrane.

In addition to Caspase-1, Caspase-4, Caspase-5, and Caspase-11, several apoptotic caspases can also induce pyroptosis. Apoptotic caspases have multiple activation mechanisms; under the influence of pathogenic microorganisms, mitochondria release apoptosis-related factors due to DNA damage, initiating intrinsic and extrinsic apoptotic signaling pathways, leading to the activation of Caspase-3 (Wang et al., 2017). Due to the presence of a natural Caspase-3 cleavage site between the N-terminal and C-terminal domains of GSDME, activated Caspase-3 cleaves GSDME at specific sites, releasing the active N-terminal domain. This N-terminal fragment then inserts into the plasma membrane, forming pores that release pro-inflammatory factors and induce pyroptosis (Bhat et al., 2023). Unlike pyroptosis, apoptosis is a form of cell death that does not involve inflammation (Kesavardhana et al., 2020). It is triggered by the activation of apoptotic caspases and can occur through both intrinsic and extrinsic pathways (Tsuchiya, 2020). The intrinsic pathway is triggered by various cellular events such as mitochondrial damage, endoplasmic reticulum stress, and reactive oxygen species formation. These stimuli lead to the release of cytochrome c (Cyt c) into the cytoplasm, which interacts with apoptotic protease activating factor-1 (Apaf-1). Cytochrome c then activates Caspase-9, which in turn activates Caspase-3/7, ultimately resulting in cell death (Hu et al., 2023). The extrinsic pathway begins with the oligomerization of death receptors on the cell surface. Once activated, Pro-caspase-8 is recruited and converted into Caspase-8, which cleaves Bid to produce tBid and activates Caspase-3/7, thus initiating a series of molecular changes that trigger apoptosis. Caspase-3 executes its function by catalyzing the cleavage of peptide bonds following aspartate residues, using a cysteine residue at its C-terminus (Zhou et al., 2018). Evidence suggests that Caspase-3 is a key linking protein in the Caspase cascade reaction (Wang et al., 2018). It can be activated through either the intrinsic or extrinsic apoptotic pathways and is the sole endogenous signal for triggering the cleavage of GSDME (Rogers et al., 2017). For decades, Caspase-3 has been recognized as a key executor of apoptosis; however, surprisingly, in the presence of GSDME, it can also participate in pyroptosis (Jiang M. et al., 2020). An increasing number of studies have found that the loss of GSDME does not inhibit Caspase-3-dependent cell death but instead alters the mode of cell death. As a substrate of Caspase-3, GSDME acts as a “conduit,” playing a pivotal role in determining whether a cell undergoes pyroptosis or apoptosis (Wang et al., 2017) (Figure 3).

#### 3.3.1 Activation of caspase through mitochondrial-dependent and anti-apoptotic protein regulatory pathways

This class of pathogens induces pyroptosis by regulating mitochondrial pathways or inhibiting anti-apoptotic proteins, leading to the activation of apoptotic caspases, such as Caspase-3. Through these mechanisms, pathogens alter the intracellular environment, promoting programmed cell death and triggering a strong inflammatory response. For example, during CAV (chicken anemia virus) infection, the viral protein Apoptin increases intracellular ROS levels, causing the aggregation of the mitochondrial membrane protein Tom20, the release of Bax and cytochrome c, which subsequently activates Caspase-9 and then Caspase-3, ultimately inducing pyroptosis (Liu et al., 2022). Following HSV-2 (Herpes Simplex Virus type 2) infection, endoplasmic reticulum stress is triggered, leading to the activation of IRE1*α*. This process results in the cleavage of the pro-apoptotic protein BID and the subsequent activation of mitochondria-dependent Caspase-3, ultimately inducing pyroptosis (Ren et al., 2023). During VSV (Vesicular Stomatitis Virus) infection, the virus inhibits the synthesis of Bcl-2 family members Mcl-1 and Bcl-xL, leading to their inactivation. This, in turn, activates downstream signaling pathways and upregulates pyroptosis-related factors, ultimately inducing pyroptosis (Orzalli et al., 2021). At its core, this group of pathogens activates Caspase-3 via mitochondrial pathways or by inhibiting anti-apoptotic proteins, leading to pyroptosis along with an inflammatory response. This underscores their essential role in modulating host cell death mechanisms.

#### 3.3.2 Activation of caspase through direct interaction with host proteins or cellular sensors

Another group of pathogens activates apoptotic caspases by directly interacting with host proteins or by triggering intracellular sensors, ultimately leading to pyroptosis. These microbes precisely regulate specific proteins to activate the inflammasome and promote cell death. For instance, during DHAV-1 (Duck Hepatitis A Virus Type 1) infection, the viral 2A2 protein directly interacts with the host MAVS protein, activating the downstream Caspase-3 signaling pathway and inducing pyroptosis (Wang et al., 2024). After ZIKV (Zika Virus) infection, the viral genomic RNA is recognized by the intracellular sensor RIG-I, which activates the RIG-I signaling pathway. This activation leads to the release of TNF-α, which in turn triggers the Caspase-8 and Caspase-3 pathways, ultimately inducing pyroptosis (Zhao Z. et al., 2022). At the core of their pathogenicity, this group of pathogens induces pyroptosis by directly interacting with host proteins or activating cellular sensors, leading to precise regulation of Caspase activation. This reflects the intricate mechanisms underlying host-pathogen interactions.

Overall, various pathogens induce pyroptosis through multiple pathways. Some rely on mitochondrial-dependent mechanisms or inhibit anti-apoptotic proteins to activate Caspase-3, triggering programmed cell death and promoting an inflammatory response. Others precisely regulate Caspase activation by directly interacting with host proteins or activating cellular sensors. These diverse mechanisms demonstrate how pathogens exploit host cell death pathways during infection to evade immune defenses or exacerbate infection. Understanding these processes provides a critical foundation for developing novel anti-infective strategies.

### 3.4 Granzyme-mediated pyroptosis pathway and its upstream regulatory mechanisms

**Figure 4:**
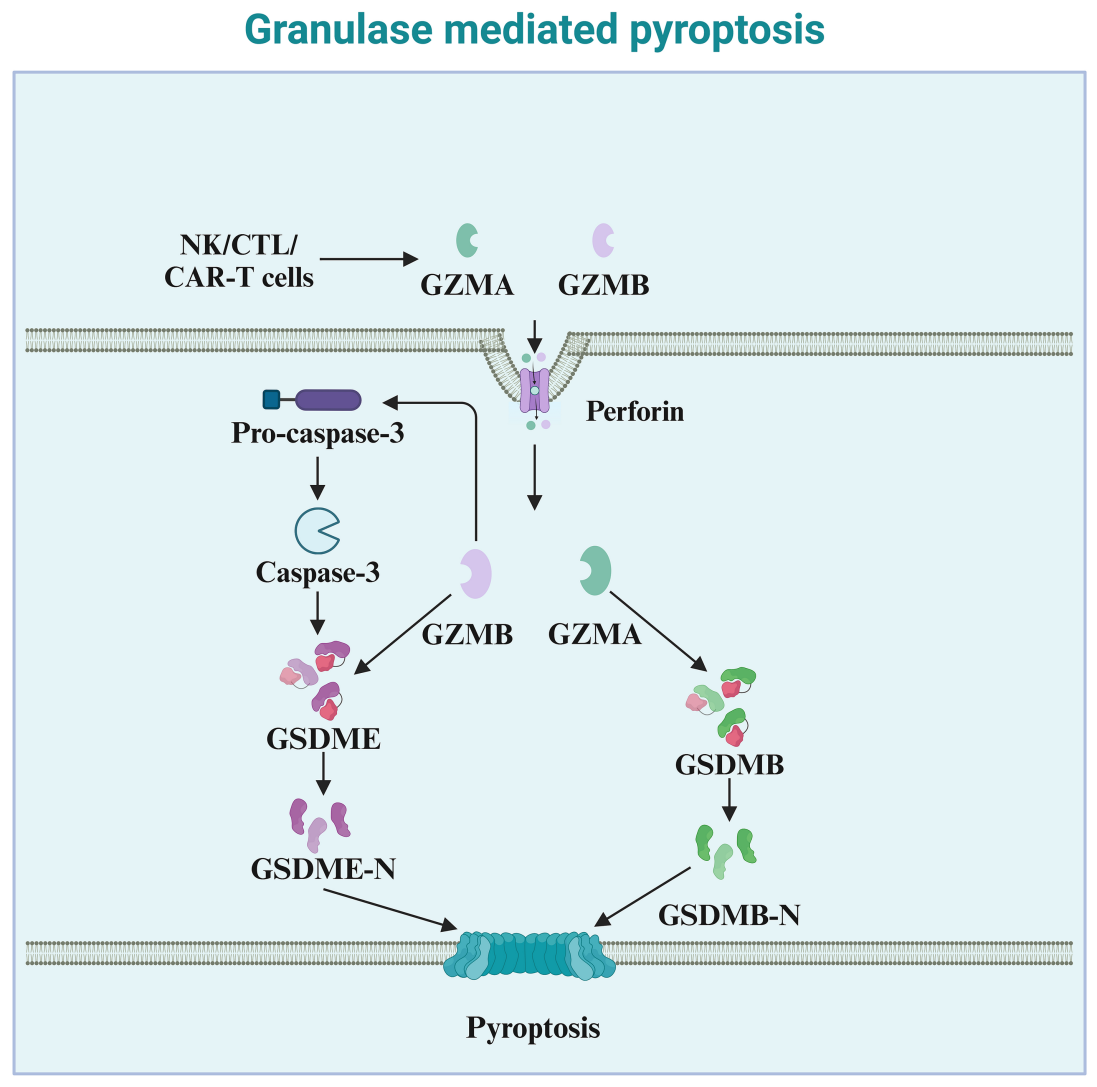
Granzyme-mediated pyroptosis. This diagram illustrates granule-mediated pyroptosis, a process involving cytotoxic immune cells such as NK cells, CTLs, and CAR-T cells. These cells release granzymes (GZMA and GZMB) and perforin to induce pyroptosis. Perforin facilitates the entry of granzymes into the target cell. Once inside the cell, GZMA and GZMB cleave Pro-caspase-3, leading to the activation of Caspase-3. Activated Caspase-3 then cleaves GSDME, producing the active GSDME-N fragment. This fragment inserts into the cell membrane, forming pores, which culminates in pyroptosis. Additionally, GZMB activates GSDMB, leading to the release of the active GSDMB-N fragment, which also contributes to membrane pore formation and pyroptotic cell death. The overall process plays a critical role in the immune defense against infected or cancerous cells.

Granzymes are exogenous serine proteases derived from the cytoplasmic granules released by cytotoxic T lymphocytes (CTLs) and natural killer (NK) cells (Zhong et al., 2023). Recent studies indicate that granzymes derived from natural killer cells, cytotoxic T lymphocytes, or chimeric antigen receptor T cells, enter target cells via perforin and can cleave specific members of the GSDM protein family, inducing pyroptosis in cancer cells. Granzyme A (GZMA), the most abundant serine protease in the granzyme family, is traditionally considered a mediator of cell death (Chowdhury and Lieberman, 2008). However, numerous reports indicate that GZMA cannot kill target cells *in vitro* unless used at very high concentrations (Metkar et al., 2008). Increasing evidence suggests a role for GZMA in regulating inflammation, such as inducing the maturation and release of pro-inflammatory cytokines. Pyroptosis, a form of cell death accompanied by the release of pro-inflammatory cytokines, may be associated with GZMA (Figure 4). An increasing number of studies have found that GZMA, derived from cytotoxic T lymphocytes, cleaves GSDMB, leading to the formation of pores on the cell membrane and inducing pyroptosis in cancer cells expressing GSDMB (Zhou et al., 2020). Therefore, the ability of GZMA to induce pyroptosis and kill cancer cells also depends on the expression of GSDMB, which is not expressed in some human tissues and is absent in mice. Natural killer cell-derived granzyme B (GZMB) can directly cleave GSDME at the same site as caspase-3, leading to the release of the effector N-terminus and penetration of the cell membrane (Zhang et al., 2020). In addition to their role in tumor immunity, granzymes also play a critical part in the immune defense against pathogens (de Jong et al., 2021). By inducing pyroptosis in infected cells, granzymes help eliminate pathogens and prevent their spread (Cigalotto and Martinvalet, 2024). NK cell-derived granzymes, in particular, are essential for controlling viral and bacterial infections through their ability to directly activate pyroptosis in infected cells (Zhao et al., 2024).

## 4 Conclusion and perspective

This article provides a brief overview of the various pathways and underlying mechanisms by which pathogenic microorganisms induce pyroptosis. The findings suggest that microorganisms trigger pyroptosis through classical inflammasome pathways, non-classical inflammasome pathways, apoptotic caspase-mediated pathways, and granzyme-mediated pathways, ultimately leading to inflammatory responses. These processes are an integral part of the body’s innate immune response, playing a crucial role in combating infections. As research on how pathogens influence pyroptosis mechanisms advances, our understanding of pyroptosis as a central component of immune defense has become more comprehensive. Moreover, pyroptosis is not merely a form of cell death; the accompanying robust inflammatory response significantly impacts the host’s defense mechanisms, offering new insights into the pathological changes that occur in the later stages of infection.

Looking forward, research on pyroptosis is expected to continue advancing our understanding of infectious and inflammation-related diseases. Further investigation in this field may reveal how pathogens evade or exploit the host’s pyroptosis response to enhance their survival and propagation. At the same time, targeted regulation of pyroptosis may emerge as a novel approach for treating infections and inflammatory diseases. A deeper understanding of these mechanisms will not only aid in the development of new anti-infective therapies but also provide a theoretical foundation for precision medicine strategies.
